# Microstructure of non-polar GaN on LiGaO_2 _grown by plasma-assisted MBE

**DOI:** 10.1186/1556-276X-6-425

**Published:** 2011-06-15

**Authors:** Cheng-Hung Shih, Teng-Hsing Huang, Ralf Schuber, Yen-Liang Chen, Liuwen Chang, Ikai Lo, Mitch MC Chou, Daniel M Schaadt

**Affiliations:** 1 Department of Physics, National Sun Yat-Sen University, Kaohsiung 80424, Taiwan; 2 Department of Materials and Opto-electronic Science, National Sun Yat-Sen University, Kaohsiung 80424, Taiwan; 3 Institute for Applied Physics/DFG-Center for Functional Nanostructures (CFN), Karlsruhe Institute of Technology, 76131 Karlsruhe, Germany

## Abstract

We have investigated the structure of non-polar GaN, both on the *M *- and *A*-plane, grown on LiGaO_2 _by plasma-assisted molecular beam epitaxy. The epitaxial relationship and the microstructure of the GaN films are investigated by transmission electron microscopy (TEM). The already reported epi-taxial relationship  and  for *M *-plane GaN is confirmed. The main defects are threading dislocations and stacking faults in both samples. For the *M *-plane sample, the density of threading dislocations is around 1 × 10^11 ^cm^-2 ^and the stacking fault density amounts to approximately 2 × 10^5 ^cm^-1^. In the *A*-plane sample, a threading dislocation density in the same order was found, while the stacking fault density is much lower than in the *M *-plane sample.

## Introduction

Gallium nitride (GaN), as one of the most important wide band semiconductors today, has far-reaching applicability in electronic and optoelectronic devices. Its hexagonal crystal structure, however, exhibits a polar axis in the [0001] direction along which a polarization is present. The resulting polarization fields lead to intrinsically existent internal electric fields which give rise to a strong quantum-confined Stark effect when group III-nitride heterostructures are grown along the [0001] direction. As a consequence, electrons and holes are spatially separated in such structures, leading to a reduced wave function overlap and a decreased radiative transition energy.

One way to circumvent these unwanted effects is to use non-polar surfaces of the hexagonal nitride structure such as the *M *-plane  and *A*-plane  for epitaxial growth procedures. The lack of available substrates for homoepitaxy on non-polar crystal planes requires alternative substrates for heteroepitaxy. While various substrates have been considered for this purpose, LiGaO_2 _(LGO) presents the unique opportunity for growth of *C *-, *M *-, and *A*-plane-oriented GaNs on a very well lattice-matched substrate, depending on the substrate surface orientation used. *C *-plane GaN growth has been demonstrated on (001) LGO by a number of groups, e.g. [1]. Recently, *M *- and *A*-plane GaN growth has been reported on (100) LGO [2] and (010) LGO [3], respectively.

In this article, we demonstrate a first analysis of *M *- and *A*-plane GaN films on LGO showing strong evidence for a high-phase purity of non-polar GaN. The TEM studies confirm the epitaxial relationship of *M *-plane GaN on (100) LGO and *A*-plane GaN on (010) LGO and give insight to their defect structure.

## Experimental procedure

The two samples discussed in this report were grown by plasma-assisted molecular beam epitaxy (PAMBE). Details on the growth of the films as well as a first structural analysis including an investigation by X-ray diffraction can be found in our previous reports in [2,3]. The *M *-plane GaN sample was grown on (100) LGO, and the *A*-plane GaN sample was grown on (010) LGO. A plan view TEM sample of the *M *-plane GaN film was prepared by mechanical polishing and subsequent Ar-ion milling. Two cross-sectional

TEM samples were fabricated for each of the GaN samples. The *M *-plane samples were cut by a focused ion beam (FIB), one looking onto the *C *-plane and one onto the *A*-plane. Mechanical polishing and Ar-ion milling were used in the preparation of the *A*-plane GaN TEM sample with the *C *-plane as the sample surface, while FIB cutting was used to produce the A-plane GaN TEM sample with the *M *-plane as the TEM sample surface. The samples were analyzed using a JEOL 3010 TEM as well as a FEI Tecnai F20 TEM, each operated with an electron acceleration voltage of 200 kV.

## Results and conclusion

**Figure 1:**
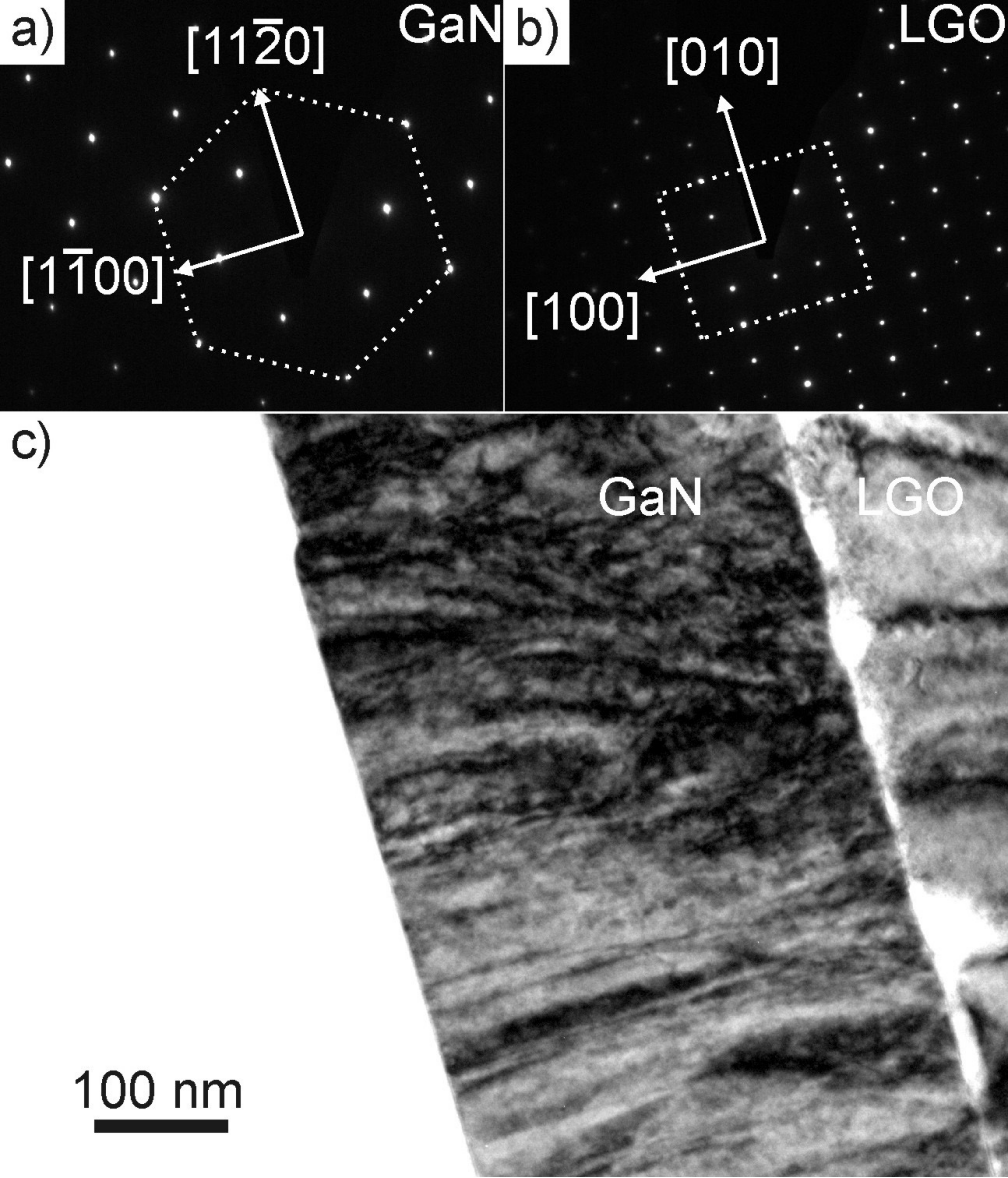
**TEM data of the *M *-plane sample, with viewing direction [0001]**. Parts **(a) **and **(b) **display the electron diffraction patterns of the GaN film and the LGO substrate seen in the bright field image of the *M *-plane GaN film **(c)**, respectively.

The epitaxial relationship of the *M *-plane GaN sample can be deduced from Figure 1a and 1b, where the diffraction patterns taken from the GaN film and the LGO substrate, seen in Figure 1c, are depicted. From the patterns, it is clear that , and . In Figure 1c, showing a bright field image of the sample cut parallel to the *C *-plane, a high density of threading dislocations is apparent. The bright areas in the substrate located directly at the interface of the substrate and the epi layer represent holes in the TEM sample caused by the electron irradiation of the transmission electron microscope. Since LGO is very sensitive to electron bombardment, it is very difficult to obtain good quality high-resolution images of this material. This issue is also discussed regarding reflection high-energy electron diffraction measurements in the growth procedure [2].

**Figure 2:**
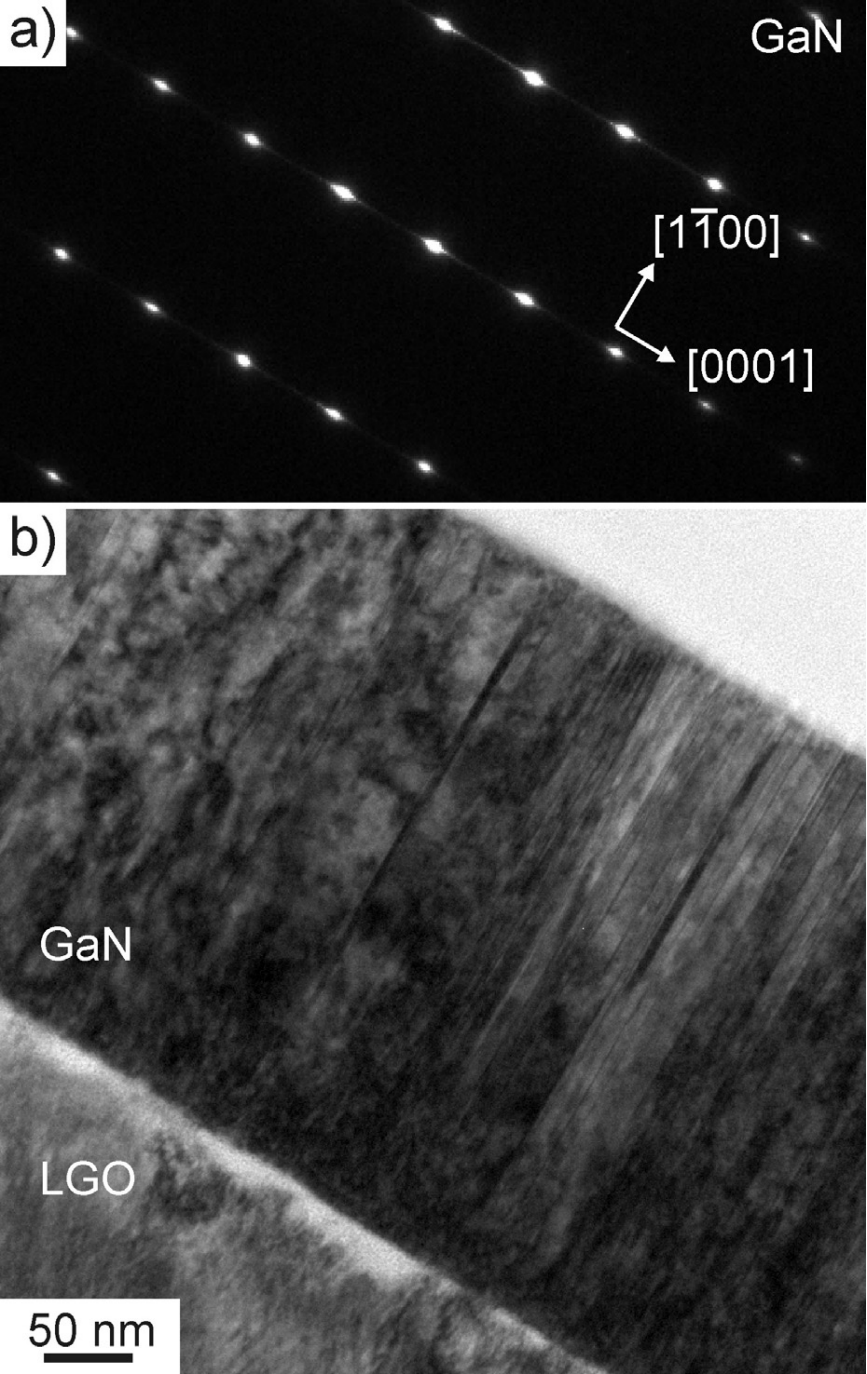
**TEM data of the *M *-plane sample, with viewing direction **. GaN diffraction pattern **(a) **and the corresponding bright field *M *-plane GaN film image **(b)**.

Taking a look at the *M *-plane GaN sample cut parallel to the *A*-plane in Figure 2a, a high density of partial dislocations associated with a high density of stacking faults can be seen. Figure 2b displays the electron diffraction pattern of the GaN film seen in Figure 2a. The diffraction spots show streaks along the [0001] direction, giving strong evidence toward a high density of stacking faults in the film.

**Figure 3:**
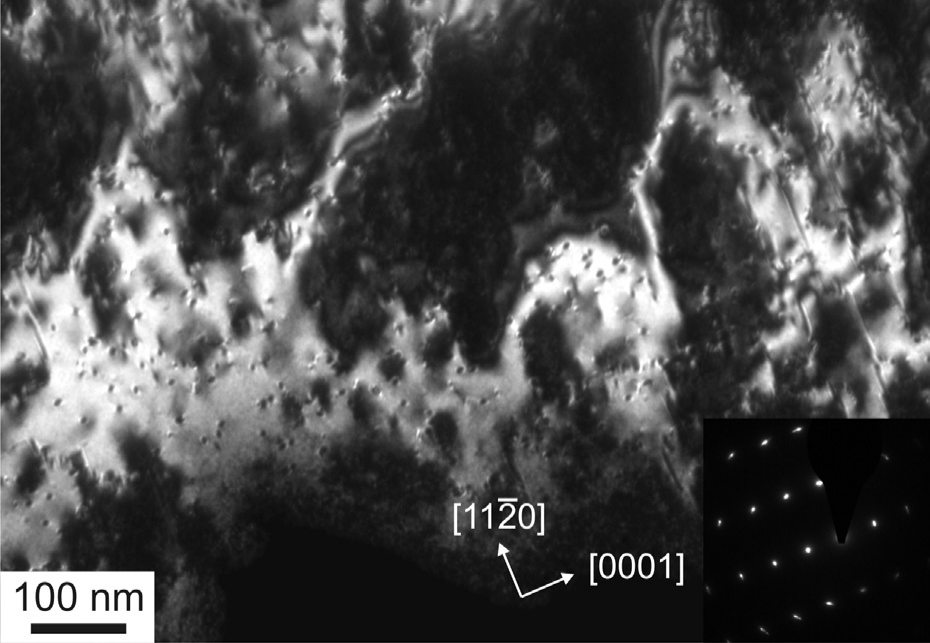
**Inplane TEM sample of the *M *-plane GaN sample**. The inset shows the GaN diffraction pattern.

The plan view TEM sample of the *M *-plane GaN film allows for an estimate of the density of threading dislocations and stacking faults. A centered dark field image of one region of the plan view sample is seen in Figure 3. The numerous small dots represent threading dislocations, while stacking faults are found running perpendicular to the [0001] direction as indicated, i.e., they lie in the *C *-plane. The elongated spots in the diffraction pattern inset in Figure 3 point toward the presence of twisted mosaic blocks in the film. The twist angle can be as high as 5°. In this sample, the threading dislocation density was found to be on the order of 1 × 10^11 ^cm^-2 ^and the stacking fault density around 2 × 10^5 ^cm^-1^. The dislocation density is higher in this sample than values reported in the literature ~ 10^9 ^cm^-2 ^[4] for *M *-plane GaN, e.g. grown on LiAlO_2 _by MOVPE. However, growth parameters had not been fully optimized on LGO and the film thickness here is twice as thin as in [4]. The thickness of the film is believed to have some impact on the threading dislocation density as is mentioned in [5] where the values for *M *-plane GaN grown on LiAlO_2 _are given as 10^9 ^cm^-2 ^near the substrate and 10^8 ^cm^-2 ^near the surface. The stacking fault density in our sample is roughly on the same order as reported elsewhere; however, values as low as 10^4 ^cm^-1 ^[4] have been reported.

**Figure 4:**
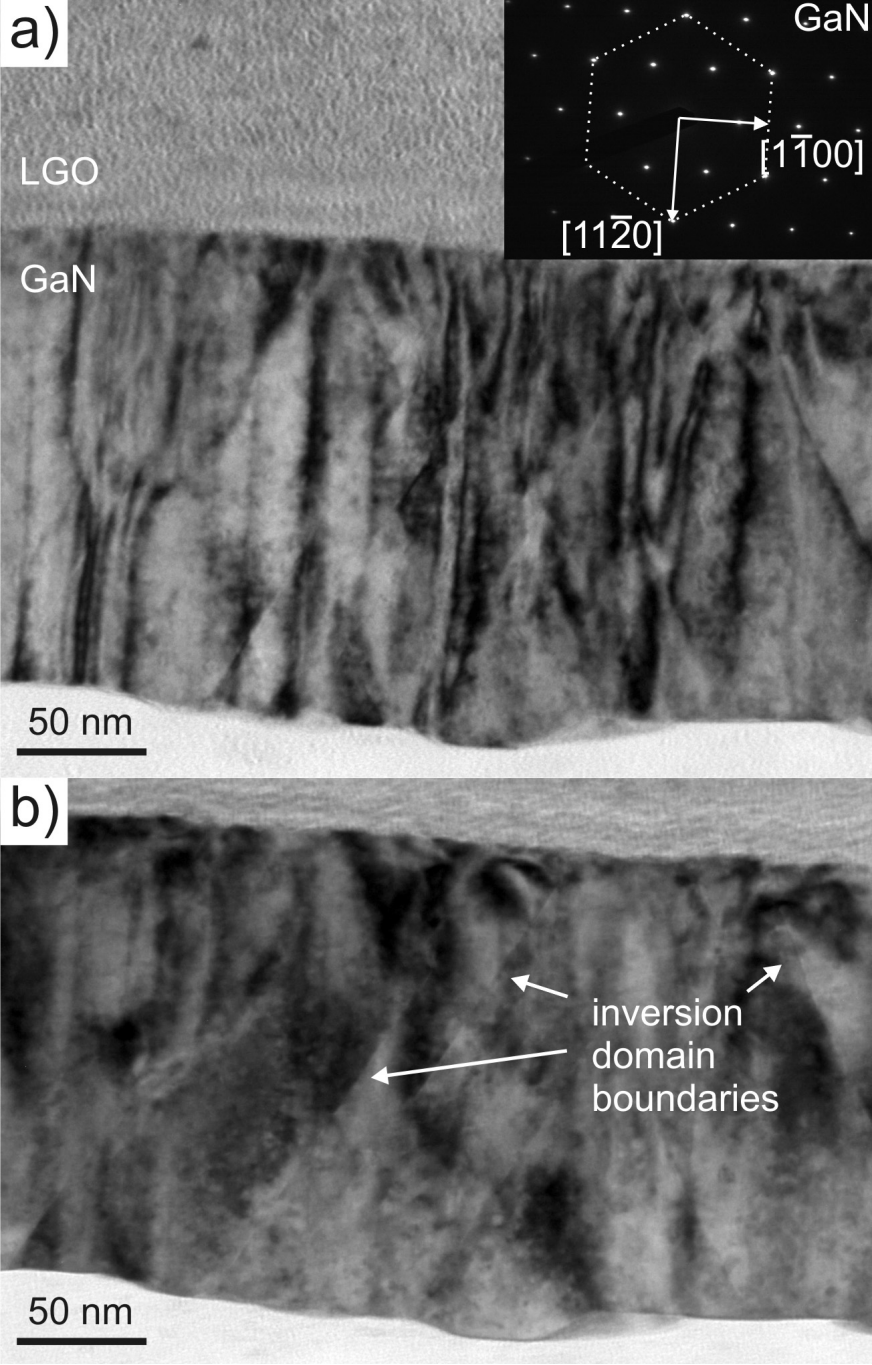
**Bright field images of the *A*-plane GaN sample taken in the two beam condition with the  (a) and  (b)**. The viewing direction for these images is [0001].

The *A*-plane GaN sample cut perpendicular to the [0001] direction, shown in Figure 4, displays a high density of threading dislocations. Comparing in-zone bright field images (not shown here) of the *M *- and *A*-plane films, the dislocation density of the two films is on the same order of magnitude, i.e., around 1 × 10^11 ^cm^-2^. The images in Figure 4a and 4b are bright field images taken in the two beam condition with the  vector parallel to the  and the  direction, respectively. The inset in Figure 4a displays the diffraction pattern of the GaN layer showing the growth of *A*-plane GaN. Dislocations that have a burgers vector parallel to  can be observed in Figure 4a; these are mixed and edge threading dislocations. In Figure 4b both pure screw and edge dislocations are out of contrast since they have burgers vectors parallel to [0001] and , respectively [6], and can there-fore not be seen. Owing to the much lower density of visible dislocations in Figure 4b , we can state that most dislocations are of either edge or screw type and a minority belongs to the mixed type. In Figure 4 inversion domain boundaries appear in both pictures as straight lines as indicated. These have an inclination of 60° with respect to the interface, i.e. they lie on the other two  planes of GaN.

**Figure 5:**
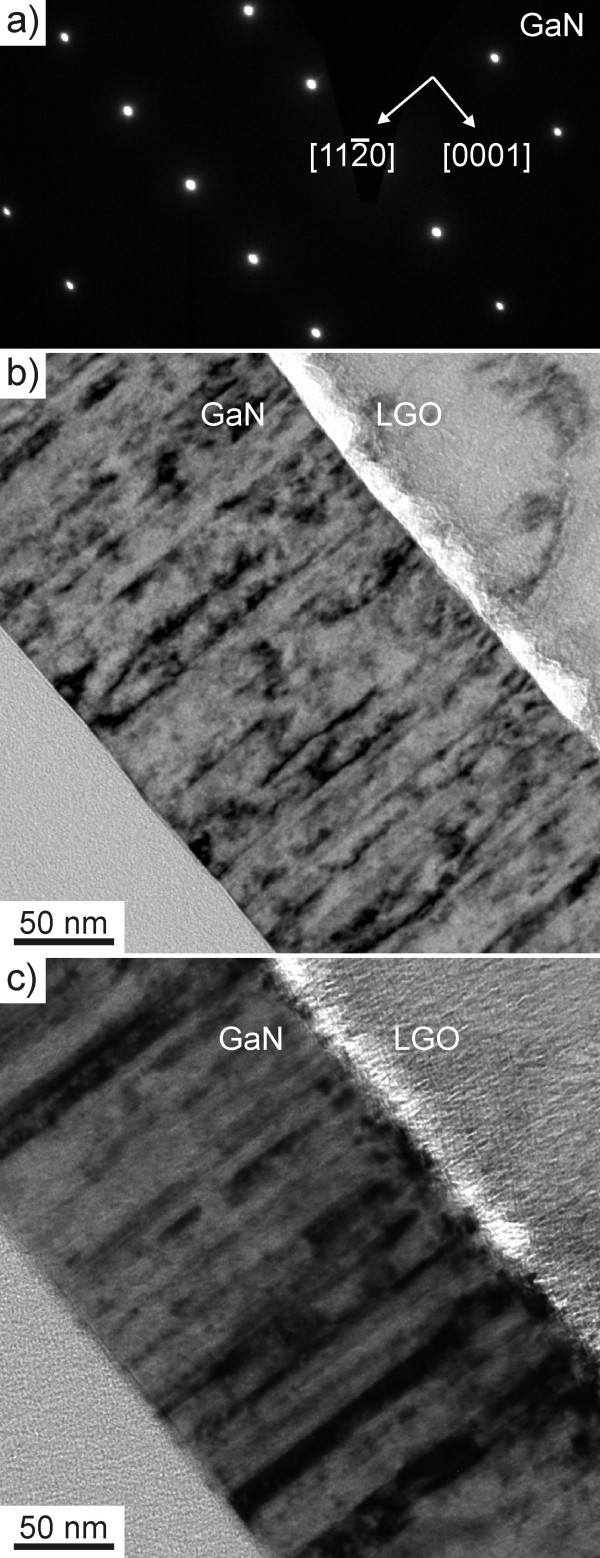
**TEM data of the *A*-plane sample, with viewing direction **. **(a) **Diffraction pattern of the GaN film of the *A*-plane GaN sample. **(b) **and **(c) **show bright field images of the same sample taken in the two beam conditions with  = (0002) and , respectively.

Figure 5b and 5c shows bright field images of the *A*-plane GaN sample cut perpendicular to the  direction taken in the two beam condition with  = (0002) and , respectively. Damage to the substrate by the electron beam is again seen. Threading dislocations as well as stacking faults are visible. Slightly more dislocations appear in the image with  = (0002) indicating that there are more pure screw threading dislocations than edge dislocations. In comparison to the *M *-plane GaN sample, much fewer stacking faults are present in the *A*-plane film. This observation is also confirmed by the missing streaks in the diffraction pattern, shown in Figure 5a. This means that the stacking fault density is lower than ~10^5 ^cm^-1^, and therefore lower than values reported previously, e.g. for *A*-plane GaN on *R*-plane Sapphire, 3.83 × 10^5 ^cm^-1 ^[7].

## Summary

*M *- and *A*-plane GaN films grown on (100) and (010) LGO, respectively, were analyzed by transmission electron microscopy. We show that the epitaxial relationship of the film deduced is in agreement with previous reports. Threading dislocations and stacking faults are the main defects in the films. For the case of the *M *-plane GaN sample, a threading dislocation density of 1 × 10^11 ^cm^-2 ^and stacking fault density of about 2 × 10^5 ^cm^-1 ^were found. The *A*-plane sample shows a threading dislocation density on the same order; however, a much lower stacking fault density is found in comparison to the *M *-plane sample.

## Abbreviations

FIB: focused ion beam; PAMBE: plasma-assisted molecular beam epitaxy; TEM: transmission electron microscopy.

## Competing interests

The authors declare that they have no competing interests.

## Authors' contributions

CHS took the TEM images with the FEI Tecnai F20 TEM. THH pre-pared the polished TEM samples and carried out the data collection with the JEOL 3010 TEM. RS carried out the growth of the samples by PAMBE, participated in preparing the samples and the taking of the TEM images, performed the analysis of the data and wrote the manuscript. YLC prepared the FIB TEM samples. LC participated in the analysis of the TEM data and conceived of the study. IL, MMCC and DMS conceived of the study. All authors read and approved the final manuscript.
